# Exploring functions for the non-lemniscal auditory thalamus

**DOI:** 10.3389/fncir.2015.00069

**Published:** 2015-11-04

**Authors:** Charles C. Lee

**Affiliations:** Department of Comparative Biomedical Sciences, Louisiana State University, School of Veterinary MedicineBaton Rouge, LA, USA

**Keywords:** auditory, thalamus, cortex, corticothalamic, thalamocortical, amygdala

## Abstract

The functions of the medial geniculate body (MGB) in normal hearing still remain somewhat enigmatic, in part due to the relatively unexplored properties of the non-lemniscal MGB nuclei. Indeed, the canonical view of the thalamus as a simple relay for transmitting ascending information to the cortex belies a role in higher-order forebrain processes. However, recent anatomical and physiological findings now suggest important information and affective processing roles for the non-primary auditory thalamic nuclei. The non-lemniscal nuclei send and receive feedforward and feedback projections among a wide constellation of midbrain, cortical, and limbic-related sites, which support potential conduits for auditory information flow to higher auditory cortical areas, mediators for transitioning among arousal states, and synchronizers of activity across expansive cortical territories. Considered here is a perspective on the putative and unresolved functional roles of the non-lemniscal nuclei of the MGB.

## Introduction

The medial geniculate body (MGB) is the main thalamic nucleus associated with audition, receiving direct synaptic inputs from the inferior colliculus (IC; Calford and Aitkin, 1983; Peruzzi et al., 1997; Crabtree, 1998; Wenstrup, 2005), thalamic reticular nucleus (TRN; Crabtree, 1998), and cerebral cortex (Winer et al., 1999, 2001), among other sources (Winer, 1992). Its projections target the cerebral cortex primarily, but also extend to subcortical sites, such as the amygdala (LeDoux et al., 1991; Bordi and LeDoux, 1994) and TRN (Crabtree, 1998; Lee and Imaizumi, 2013). Classically, the MGB can be divided into three main divisions based on cytoarchitectural, connectional, and physiological criteria (Calford and Aitkin, 1983; Calford, 1983; Imig and Morel, 1985; Clerci and Coleman, 1990; Hashikawa et al., 1991; Smith et al., 2012; Imaizumi and Lee, 2015): the ventral (MGBv), dorsal (MGBd), and medial (MGBm) divisions (Winer, 1984; Rouiller et al., 1989); although, further subdivisions are proposed in some species, particularly within the dorsal division (Jones, 2007; Lee and Winer, 2011b).

Among these MGB divisions, the principal, or lemniscal nucleus, the ventral division (MGBv) receives topographically organized projections from the central nucleus of the IC and projects to tonotopically-organized areas of the auditory cortex (AI; McMullen and de Venecia, 1993; Lee et al., 2004a; de la Mothe et al., 2006b; Lee and Winer, 2008a; Llano and Sherman, 2008; Razak and Fuzessery, 2010; Hackett et al., 2011; Smith et al., 2012). In contrast, the dorsal division nuclei are non-tonotopically organized and are connectionally affiliated with corresponding non-tonotopically organized regions of the midbrain (dorsal division of the IC; ICd) and auditory cortex (e.g., secondary auditory cortex (AII); Huang and Winer, 2000; Smith et al., 2012). Finally, and perhaps most enigmatic, the medial division of the MGB receives polymodal inputs from the IC and projects broadly across many tonotopic, non-tonotopic, multimodal and limbic cortical areas (Lee and Winer, 2008a; Imaizumi and Lee, 2015), terminating notably in cortical layers 1 and 6 (Huang and Winer, 2000) and also in the amygdala (LeDoux et al., 1991).

While the physiological properties of the tonotopic ventral division of the MGB have been intensively investigated (Aitkin and Webster, 1972; Calford and Webster, 1981; Imig and Morel, 1985; Morel and Imig, 1987; Miller et al., 2001, 2002), similar studies of the non-lemniscal MGB nuclei in relation to the ventral division are ongoing (Aitkin, 1973; Calford and Aitkin, 1983; Calford, 1983; Rouiller et al., 1989; Bordi and LeDoux, 1994; Bartlett and Smith, 1999; Edeline et al., 1999; Wenstrup, 1999; He and Hu, 2002; He, 2002; Anderson et al., 2007; Anderson and Linden, 2011; Bartlett and Wang, 2011). Indeed, this is not unique to the auditory system, as the roles of non-primary thalamic nuclei in other systems have generally not been well defined (Sherman and Guillery, 2006; Jones, 2007; Cruikshank et al., 2012). However, we have suggested that some of these non-primary nuclei likely have important roles in the transfer of information to higher auditory cortical centers (Lee and Sherman, 2010a, 2011), while others likely are involved in emotive and affective processing of auditory information (Iwata et al., 1986; Weinberger, 2011). Modern experimental approaches will likely shed light on those thalamic nuclei whose functions have yet to be defined (Cruikshank et al., 2012).

## Non-Lemniscal Auditory Thalamic Nuclei as Information-Bearing Conduits

**Figure 1:**
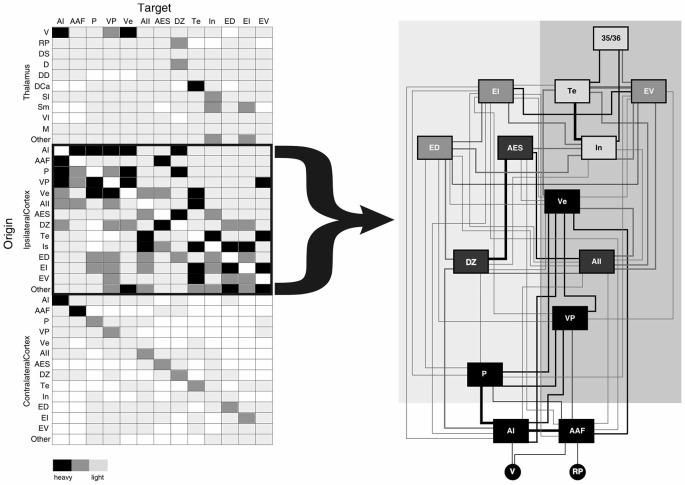
**Schematic summary of convergent projections from thalamic and cortical sources to the thirteen areas of the cat auditory cortex (AI).** Left panel depicts a simplified schematic of the relative input arising from each thalamic and cortical source based on the percent input to each area. Right panel depicts a serial hierarchical ordering of auditory cortical areas in the cat based on the laminar origins of cortical projections. Such a cortical hierarchy (right panel) reflects the notion that the numerous corticocortical projections (left panel) are the main determinants of information processing in higher cortical areas. Neglected in this view are roles for the several non-primary thalamocortical (as well as the commissural cortical) inputs to each auditory area, which also contribute fewer, yet potentially salient convergent information to each area. Shading intensity on left panel depicts relative strength of inputs (heavy, medium, weak). Area box shading in right panel indicates type of auditory area (black = tonotopic, dark gray = non-tonotopic, medium gray = polymodal association, light gray = limbic) and line weights reflect average connectional strength indicated in left panel. Figure adapted from Lee and Winer (2011a). Abbreviations: AAF, anterior auditory field; AES, anterior ectolsylvian sulcal area; AI, primary auditory area; AII, second auditory cortex; D, dorsal division of the medial geniculate body (MGB); DCa, dorsal caudal nucleus of the MGB; DD, deep dorsal nucleus of the MGB; DS, dorsal superficial division of the MGB; DZ, dorsal auditory zone; ED, dorsal posterior ectosylvian area; EI, intermediate posterior ectosylvian area; EV, ventral posterior ectosylvian area; In, insular cortical area; M, medial division of the MGB; MGB, medial geniculate body; P, posterior auditory area; RP, rostral pole of the MGB; Sl, lateral suprageniculate nucleus; Sm, medial suprageniculate nucleus; Te, temporal auditory area; V, ventral division of the MGB; Ve, ventral auditory area; Vl, vetrolateral nucleus of the MGB; VP, ventroposterior auditory area.

Many thalamic and cortical projections converge in each auditory cortical area, with the most numerous extrinsic inputs arising from other ipsilateral cortical areas (~80% of the total extrinsic input to each auditory area in the cat, Figure 1; Lee and Winer, 2011a). Similar connectional patterns organize auditory regions in many mammalian species, including the monkey (Hackett et al., 1998; de la Mothe et al., 2006a,b), cat (Lee and Winer, 2008a,b,c), bat (Fitzpatrick et al., 1998), rat (Roger and Arnault, 1990; Shi and Cassell, 1997), mouse (Llano and Sherman, 2008; Oh et al., 2014; Takemoto et al., 2014), ferret (Bizley et al., 2005), and gerbil (Budinger et al., 2000; Takesian et al., 2012). Due to the preponderance of such corticocortical convergence, hierarchical cortical models form the basis for many connectional frameworks linking these auditory areas (Figure 1; Rouiller et al., 1991; Kaas and Hackett, 2000; Hackett, 2011; Lee and Winer, 2011a,b), similar to those proposed for the visual and somatosensory systems (Felleman and Van Essen, 1991).

The role of the thalamus has generally been disregarded in these hierarchical cortical models beyond that of the primary thalamic nuclei and instead the non-primary nuclei are often assigned a modulatory role (Olshausen et al., 1993). Canonically then, auditory information is often viewed as ascending through the central auditory lemniscal pathway from the cochlea through the brainstem, midbrain (IC), and thalamus (MGBv) until it reaches the primary auditory area and is subsequently processed through the copious corticocortical network (Figure 1). However, as we have noted above, every auditory cortical area receives some fraction of its convergent input from the thalamus (~10% of the total extrinsic input, Figure 1; Lee and Winer, 2008a, 2011a,b). Why then should these non-lemniscal thalamic inputs to higher auditory cortical areas have no role in auditory information processing?

**Figure 2:**
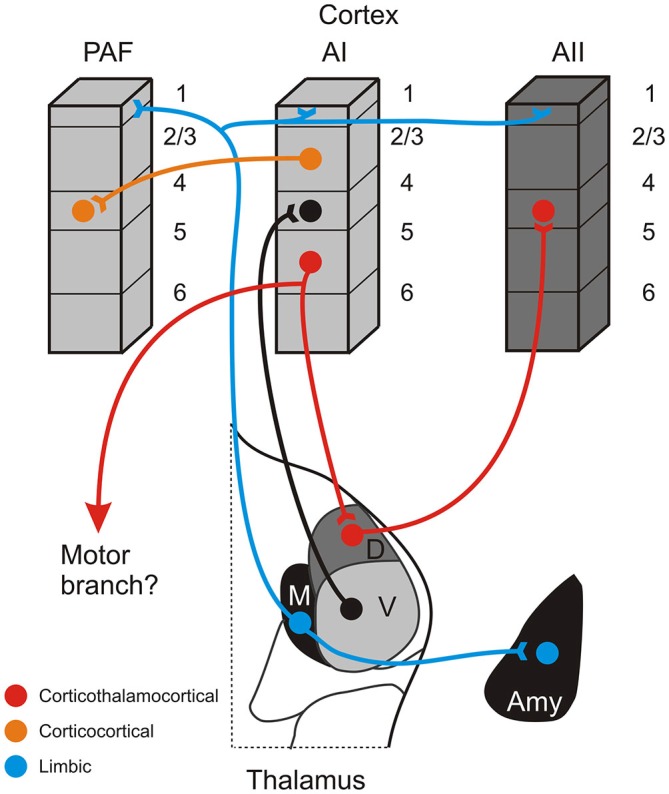
**Schematic summary of some connections of the non-lemniscal auditory thalamus.** The red pathway depicts a potential information-bearing route linking cortical areas via a corticothalamocortical pathway originating in layer 5 of a lower cortical area (AI), synapsing in a higher-order thalamic nucleus (D), and projecting to layer 4 of a higher cortical area (AII). The same layer 5 CT neurons may also branch to innervate lower motor centers. This pathway is distinct from the more numerous direct corticocortical connections that link many auditory areas, such as AI and the posterior auditory field (PAF), depicted by the orange pathway. A complementary system, putatively involved in affective processing of auditory information and synchronizing activity across cortical territories, is depicted by the blue pathway, which has widespread terminations in layer 1 of multiple areas and the amygdala (Amy). Omitted from the figure for simplicity are the projections of the medial division to layer 6 and also feedback CT projections originating in layer 6 of each area.

Indeed, we have previously argued that, despite their relative minority, these higher-order auditory thalamocortical connections provide an important alternate conduit for conveying information between cortical areas via a *corticothalamocortical* route (Figure 2: red pathway; Lee and Sherman, 2010a, 2011). This route originates from layer 5 of a lower-order auditory cortical area (e.g., AI) and terminates non-reciprocally in a higher-order thalamic nucleus (e.g., MGBd; Bartlett et al., 2000; Huang and Winer, 2000; Llano and Sherman, 2008). These layer 5 neurons potentially may branch to innervate motor centers, serving as an efference copy of motor signals to higher auditory centers, as has been similarly suggested for the visual and somatosensory systems (Guillery, 2003; Sherman and Guillery, 2011). The superior colliculus may be the most likely target for such an auditory efference copy (Harting et al., 1992; Chabot et al., 2013), as similar branching of layer 5 CT neurons appears absent to the IC (Wong and Kelly, 1981; Lee et al., 2011).

Completing this circuit, the higher-order thalamic nucleus then projects to a higher order auditory cortical area (e.g., AII; Figure 2: red pathway; Lee and Sherman, 2008; Llano and Sherman, 2008). Neuronal projections along this alternate corticothalamocortical route have anatomical and physiological properties suited for high-fidelity neuronal conduits for information processing in the nervous system, which we have previously termed “driver” or “class-1” pathways (Lee and Sherman, 2010a, 2011). The driver-like projections typically exhibit thick axons with giant terminal endings and depressing synapses that activate only ionotropic glutamate receptors (iGluRs; Lee and Sherman, 2010a, 2011). Thus, for example, the layer 5 corticothalamic pathway from AI exhibits thick axons that end in giant terminals in the dorsal division of the MGB (Ojima, 1994; Winer et al., 1999; Llano and Sherman, 2008). Such synapses then, despite their numerical minority, can exert a potent influence on their postsynaptic targets, much like the numerically sparse retinogeniculate projection (Sherman and Guillery, 1998; Winer et al., 1999; Bartlett et al., 2000; Llano and Sherman, 2008). In addition, the thalamocortical pathways from MGBv and MGBd to AI and AII, respectively, both exhibit driver-like, high-probability of release synapses, characterized by a depressing response to paired stimulation that activates only iGluRs, that while weak individually, are highly-reliable and can synchronize to drive receptive field formation in the cortex (Rose and Metherate, 2005; Bruno and Sakmann, 2006; Lee and Sherman, 2008). As such, in this framework, higher-order auditory thalamic nuclei, like the MGBd, are proposed as driver-like conduits for information flowing from lower auditory cortical areas to higher auditory cortical areas (Figure 2: red pathway).

We have demonstrated the plausibility of such a corticothalamocortical conduit, both anatomically and physiologically, for very early stages of the auditory cortical pathway in mice (i.e., AI-MGBd-AII; Lee and Sherman, 2008, 2009, 2010b); however, it is still unknown the extent to which these corticothalamocortical pathways are linked beyond these areas (Lee and Sherman, 2011). Still, it appears likely that the anatomical substrates exist for corticothalamocortical pathways to link all auditory cortical areas (Winer et al., 1999, 2001; Huang and Winer, 2000; Smith et al., 2012). In particular in the cat, the giant, driver-like corticothalamic terminals originate from all auditory cortical areas and target various nuclei in the dorsal MGB (e.g., dorsal nucleus, dorsal superficial nucleus, deep dorsal, etc.; Winer et al., 1999), which in turn project to layer 4 of several auditory cortical areas (Huang and Winer, 2000; Lee and Winer, 2008a). Interestingly, giant corticothalamic projections originating from different areas may target the same thalamic nucleus, such as the projections to MGBd from areas AI, AAF, Ins, and AII in the cat (Winer et al., 1999) or areas AI and AAF in the mouse (Llano and Sherman, 2008), establishing potential hubs for convergent information processing in the thalamus. Indeed, such convergent corticothalamic geometries are perhaps more parsimonious with the notion of each thalamic nucleus and cortical area forming units of degenerate, web-like, processing ensembles (Lee and Winer, 2011a,b), rather than strictly limited by serial hierarchical processing networks (Felleman and Van Essen, 1991). However, defining the precise nature of these corticothalamocortical routes through each thalamic nucleus and auditory cortical area will require further neuroanatomical and physiological studies.

## Corticocortical vs. Corticothalamocortical

An open question here is the manner in which different auditory areas interact, whether via the direct corticocortical route, the indirect corticothalamocortical route, or a combination of both routes (Figure 2: red vs. orange pathways; Felleman and Van Essen, 1991; Rouiller et al., 1991; Lee and Winer, 2011b). Although we have previously posited that this alternate route exists between AI and AII (Lee and Sherman, 2008, 2010b, 2011), it remains unclear the extent to which these alternate corticothalamocortical pathways prevail throughout the auditory forebrain. That is, are certain cortical areas preferentially linked via corticocortical or corticothalamocortical connections? What benefits accrue to information processing via these types of pathways? How are these routes organized globally across all auditory areas? Of course, these issues are unresolved, but some connectional observations may be pertinent to deciphering them.

In general, groups of physiologically similar areas are related by their forebrain connections. This principle is particularly evident in the monkey (Hackett et al., 1998; Kaas and Hackett, 2000), where auditory areas are grouped into core, belt and parabelt regions based on connectivity and physiology (de la Mothe et al., 2006a,b). In the cat, tonotopic areas are preferentially linked by their cortical and thalamic inputs, while the non-tonotopic, association and limbic areas likewise each have distinct connectional affiliations (Lee et al., 2004a,b, 2011; Lee and Winer, 2005, 2008a,b,c, 2011a,b; Lee, 2013).

However, physiologically different areas generally have much sparser direct corticocortical connections (Fitzpatrick et al., 1998; Budinger et al., 2000; Bizley et al., 2005; de la Mothe et al., 2006a; Lee and Winer, 2008c). For example, in the cat, similar areas, such as AI and posterior auditory field (PAF), are linked via numerous corticocortical and thalamocortical connections (Figure 1; Lee et al., 2004a; Lee and Winer, 2008c). Both of these areas receive direct inputs from the ventral division of the MGB (MGBv) to which they send feedback reciprocal corticothalamic projections that originate in layer 6 (Winer et al., 2001; Lee and Winer, 2008a). But, in comparison, physiologically dissimilar areas, the primary (AI) and secondary auditory cortices (AII), are weakly interconnected by corticocortical and thalamocortical connections (Figure 1; Lee and Winer, 2008c, 2011a).

How then might information be transferred between these auditory cortical areas in the cat: AI, P, and AII? Conjecturing based on the connectivity in the cat, we would suggest that the alternate *corticothalamocortical* route may preferentially transfer information between the physiologically dissimilar areas (tonotopic and non-tonotopic), AI and AII, via layer 5 of AI to MGBd and then to layer 4 of AII (Figure 2: red pathway; Lee and Sherman, 2008, 2010a, 2011). On the other hand, the *corticocortical* route might be utilized preferentially for transferring information between physiologically similar (tonotopic) areas, such as AI and PAF (Figure 2: orange pathway; Morel and Imig, 1987; Lee and Winer, 2008a).

By comparison, in primates, connections among areas with similar physiological properties (e.g., core area connections) also tend to be greater than inter-group connections (e.g., core to belt area connections), although the magnitude of these inter-group connections seems greater in primates compared with cats (Hackett et al., 1998; Kaas and Hackett, 1998; de la Mothe et al., 2006a, 2012). It is plausible, therefore, that species-specific constraints govern the degree to which corticocortical and corticothalmocortical pathways are utilized, perhaps akin to the species-specific evolutionary trade-offs in the MGB that differ in their utilization of interneuronal or reticulothalamic inhibitory inputs (Winer and Larue, 1996).

However, rather than forming the basis of a strict prediction, one might better approach these conjectures as a framework for deciphering future physiological investigations to consider both the corticocortical and corticothalamocortical routes as potential neural substrates in auditory forebrain operations. The question then of utility of these two pathways in auditory forebrain operations might be better construed as one of degree, rather than that of hegemony.

## The Medial Division of the MGB

A caveat to this notion of the non-lemniscal MGB nuclei as conduits for information flow to higher auditory cortical areas is the medial division of the MGB. Unlike the nuclei of the dorsal division, the medial division does not appear to be a major nuclear target of the giant, driver-like corticothalamic projections that establish the first leg of the corticothalamocortical pathway (Figure 2; Winer et al., 1999; Llano and Sherman, 2008). Furthermore, unlike both the ventral and dorsal divisions, the medial division does not project specifically to one or a few cortical areas, but rather projects broadly across nearly all auditory cortical regions, terminating prominently in cortical layer 1, rather than the classical thalamic input layer 4 (Huang and Winer, 2000; Jones, 2003; Lee and Winer, 2008a; Llano and Sherman, 2008; Smith et al., 2012). As such, the neuroanatomical substrates supporting the corticothalamocortical pathway, as initially formulated, appear to be lacking for the medial division of the MGB, but see Cruikshank et al. (2012) for a consideration of similar thalamic projections in the prefrontal cortex.

Instead then, the prevailing notion for the medial division of the MGB considers it to be part of the matrix system of thalamic nuclei, proposed by Jones (2001) in his core-matrix model of thalamic organization. In this framework, thalamic nuclei are distinguished on the basis of the expression of different calcium binding proteins, i.e., parvalbumin is highly expressed in the core thalamic cells, as in the ventral division of the MGB, while calbindin is expressed strongly in the matrix cells, as in the medial division of the MGB (Hashikawa et al., 1991; Molinari et al., 1995; Jones, 2001, 2003; Lu et al., 2009). The thalamocortical projection patterns of these cell types are similarly distinct, with the core thalamic neurons projecting to layer 4 in specific areas, while the matrix neurons project diffusely across the cortex, targeting layer 1 and potentially different classes of excitatory projection neurons (Figure 2: blue lines; Hashikawa et al., 1991; Molinari et al., 1995; Jones, 2001; Harris and Shepherd, 2015). Likewise, the functions for these two systems are proposed to be distinct, with the core thalamocortical system analogous to the first and higher-order pathways discussed above, while the matrix thalamocortical system, of which the medial division is a part, likely exerts control over broad cortical territories, possibly regulating excitability and synchronizing activity in response to different behavioral arousal states (Mitani and Shimokouchi, 1985; Hipp et al., 2011). Yet, this parcellation alone does not capture the full complexity of the auditory thalamus, since the dorsal division, in part, may also be considered part of the matrix system (Hashikawa et al., 1991; Molinari et al., 1995; Jones, 2001, 2003; Lu et al., 2009). Thus, additional neuroanatomical and physiological features must further distinguish the unique roles of the medial division from those of the dorsal and ventral division (LeDoux et al., 1985, 1991; Iwata et al., 1986; Cruikshank et al., 1992).

In this regard, the connections of the medial division of the MGB with the limbic-related nuclei in the amygdala position it uniquely to alter auditory forebrain networks in affective and emotional responses to aversive stimuli (Figure 2: blue line; LeDoux et al., 1985, 1991; Iwata et al., 1986; Cruikshank et al., 1992). The same regions of the amygdala also receive descending convergent inputs from the AI (Romanski et al., 1993), which may in turn affect other auditory cortical areas (McDonald and Jackson, 1987; Miyashita et al., 2007), perhaps establishing essential circuits for synchronizing and coalescing auditory forebrain ensembles in response to salient affective stimuli (Winer, 2006; Weinberger, 2011). Moreover, due to its central position in the distribution of afferent information to both the amygdala and cortex, the medial division of the MGB may act as the central hub for auditory fear conditioning (Weinberger et al., 1995; Weinberger, 2011).

Overall though, it is clear that the operations of the non-lemniscal medial and dorsal division nuclei of the MGB extend and enhance the operations of the auditory thalamus beyond that of a simple relay for acoustic information entering the auditory cortical network. The ultimate challenge for future investigations will be to specifically parse their interrelated roles in global auditory forebrain processes and the emergent construction of holistic auditory percepts.

## Conflict of Interest Statement

The author declares that the research was conducted in the absence of any commercial or financial relationships that could be construed as a potential conflict of interest.
